# Impact of Prior Diabetic Retinal Screening on Hospitalization and Ophthalmic Follow-Up in Diabetic Patients with Newly Diagnosed Proliferative Diabetic Retinopathy

**DOI:** 10.3390/diagnostics16101562

**Published:** 2026-05-21

**Authors:** Charles Zhang, Neel R. Sonik, Zoe J. Tsoukas, Jonathan B. Lin, Georges AbouKasm, Jason C. Fan, Ninel Z. Gregori

**Affiliations:** 1 Bascom Palmer Eye Institute, Department of Ophthalmology, Miller School of Medicine, University of Miami, 900 NW 17th Street, Miami, FL 33136, USA; ngregori@med.miami.edu; 2 Department of Ophthalmology, Ross Eye Institute, State University of New York at Buffalo, Buffalo, NY 14203, USA; 3 Miller School of Medicine, University of Miami, Miami, FL 33136, USA; nsonik@med.miami.edu (N.R.S.); zjt17@miami.edu (Z.J.T.); gaboukasm@miami.edu (G.A.); 4 Department of Ophthalmology, Byers Eye Institute, Stanford University School of Medicine, Palo Alto, CA 94303, USA; jblin@stanford.edu; 5 Department of Ophthalmology, Duke University, Durham, NC 27710, USA; jason.fan@duke.edu; 6 Miami Veterans Affairs Medical Center, Miami, FL 33125, USA

**Keywords:** diabetes complications, diabetic retinopathy, electronic health records, follow-up, healthcare utilization, hospitalization, loss to follow-up, proliferative diabetic retinopathy, propensity score matching, screening

## Abstract

**Background/Objectives**: This retrospective cohort study compared hospitalization and follow-up rates in patients with newly diagnosed proliferative diabetic retinopathy (PDR) versus those without prior diabetic retinopathy (DR) screening. **Methods:** Using TriNetX, a global electronic health record database, 57,964 patients aged ≥ 40 years with type 2 diabetes and newly diagnosed PDR without diabetic macular edema (DME) requiring panretinal photocoagulation or intravitreal injection were included. Patients were stratified based on the presence or absence of prior DR screening in the last 5 years and balanced using propensity score matching (PSM). Primary outcomes included 30-, 60-, and 90-day hospitalization rates and repeat ophthalmic follow-up as estimated using repeat PDR diagnosis codes and repeat retinal imaging codes, including OCT, fundus photography, and fluorescein angiography. **Results:** Of 57,964 patients, 25,003 had no prior DR screening and 32,961 had prior DR screening. After matching, 19,316 patients were included per cohort. Patients without known DR screening had significantly higher hospitalization rates at 30 days (RR = 1.78, 95% CI 1.67–1.89), 60 days (RR = 1.59, 95% CI 1.51–1.67), and 90 days (RR = 1.51, 95% CI 1.44–1.58), and lower repeat ophthalmic visits by PDR codes at 30 days (RR = 0.458, 95% CI 0.440–0.476), 60 days (RR = 0.450, 95% CI 0.437–0.463) and 90 days (RR = 0.420, 95% CI 0.408–0.432) or by repeat retinal imaging codes at 30 days (RR = 0.450, 95% CI 0.423–0.478), 60 days (RR = 0.394, 95% CI 0.377–0.411), and 90 days (RR = 0.381, 95% CI 0.366–0.396) (all *p* < 0.0001). **Conclusions:** Absence of known prior DR screening in PDR patients is associated with higher hospitalization risk and reduced ophthalmic follow-up, suggesting that a lack of screening indicates broader gaps in healthcare engagement and disease control. Tailored strategies are needed to prevent vision loss as well as systemic complications.

## 1. Introduction

Diabetic retinopathy (DR) is the leading cause of blindness among working-age adults in the US [1,2]. Proliferative diabetic retinopathy (PDR) represents the most advanced form, which can lead to complications such as vitreous hemorrhage (VH), tractional retinal detachment (TRD) and neovascular glaucoma (NVG) [3,4]. Treatment of PDR can be performed using panretinal photocoagulation (PRP), which has been demonstrated to reduce the risk of severe vision loss by 50% [5]. More recently, anti-vascular endothelial growth factor (VEGF) therapy was demonstrated to be non-inferior to PRP [6,7]. However, these results are based on controlled clinical trial settings with intensive patient tracking and follow-up. Real-world studies have shown significant loss to follow-up among diabetics—particularly in younger, lower income and minority populations—and worse anatomic and functional outcomes in eyes treated with anti-VEGF only versus those that received PRP [2,8,9].

In routine clinical practice, delivering PDR care is challenging due to poor patient adherence and systemic health factors that could limit follow-up. Many patients with diabetes have multiple comorbidities, socioeconomic constraints and healthcare access issues [10,11,12]. A recent study reported that roughly 25% of PDR patients failed to return after treatment with PRP or anti-VEGF [8]. Patients that are lost to follow-up (LTFU) may experience recurrence of their disease, with it often occurring at an earlier time point in those receiving anti-VEGF injection [13,14]. Therefore, LTFU is an important factor to consider when extrapolating clinical trial outcomes into the real-world.

While guidelines advise annual dilated fundus examinations for patients with diabetes, screening rates have reached as low as 60% [2,15]. Patients who do not undergo routine screening may initially present with more advanced stages of DR [16]. Furthermore, the presence of PDR is correlated with higher rates of nephropathy, neuropathy and cardiovascular disease, and is representative of longstanding, poorly controlled systemic disease [17,18]. Thus, patients initially presenting with PDR without a prior history of DR exams are likely to have undiagnosed comorbidities associated with diabetes. These may predispose them to acute complications such as cardiovascular events, infections and metabolic diseases that may require hospitalization, which, in addition to economic and social factors, may limit their ability to return for ophthalmic care [19].

Thus, the current study aims to investigate real-world outcomes in patients with newly diagnosed PDR. Specifically, this study aims to examine the influence of prior DR screening on hospitalization risk and ophthalmic follow-up. Understanding these relationships will help clinicians better understand the risk of LTFU to improve patient counseling and treatment choices for PDR.

## 2. Materials and Methods

The current study is a retrospective cohort study using the TriNetX Analytics Network, a federated network of de-identified electronic health records from numerous healthcare organizations. This platform uses aggregate clinical data across a diverse set of international institutions, both academic and non-academic. The study was exempt from institutional review board approval as only de-identified data were analyzed. This study was conducted in accordance with the principles of the Declaration of Helsinki and followed the Strengthening the Reporting of Observational Studies in Epidemiology guidelines for reporting cohort studies [20].

### 2.1. Study Population

Data from 2010 to 2024 were collected from the TriNetX Health Research Network on 12 June 2025. TriNetX contains diagnoses codes using International Classification of Disease, Tenth Revision, Clinical Modification code set (ICD10-CM). Additionally, Current Procedural Terminology (CPT) and Systematized Nomenclature of Medicine Clinical Terms (SNOMED) codes were utilized where applicable. Full details of coding data are provided in Supplementary Table S1. Patients ≥ 40 years with type 2 diabetes mellitus (ICD-10 code E11.X) were included. The age cutoff of 40 years was selected because the average age of diagnosis of type 2 DM is 30–40 years [21]. Additionally, to capture miscoded patients, patients over the age of 40 with a hemoglobin A1c ≥ 6.5% (represents the diagnosis of diabetes under any circumstance) without a history of Type 1 DM (E10.X) or other secondary DM (E13.X) were included if they met the PDR criteria below.

Diagnosis of PDR without DME due to type 2 DM was chosen as our study entry criteria (E11.352, E11.353, E11.354, E11.355, E11.359). The index event was set as the first instance of PRP (67228) or intravitreal injection (67028) for that ICD-10 code in the outpatient setting. Patients with type 2 DM with ophthalmic complications (E11.3) and VH (H43.1) that were managed with an intravitreal injection (67028) in the absence of an alternative reason for VH (H43.81, S05.X, H34.81, H34.82) were also included.

**Figure 1:**
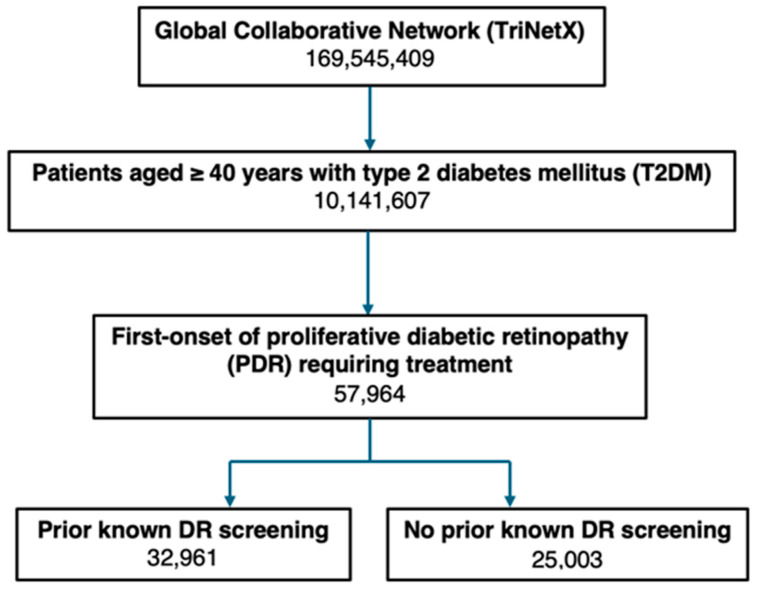
Selection and stratification of patients with PDR and known prior DR screening and controls without known prior DR screening.

Patients were categorized based on evidence of prior DR screening using ophthalmology-specific encounter codes or indicators of an ophthalmic exam within the preceding 5 years. Prior DR screening was selected as a proxy for preventative care engagement to reflect real-world conditions at the time of PDR diagnosis. Eye care providers typically will only have access to prior ophthalmic screening information within their own clinical systems. This was evaluated by the previous use of a new or established patient eye code (CPT 92002, 92004, 92012 or 92014), fundus photography (CPT 92250) or optical coherence tomography (CPT 92134), or a previous diagnosis of mild NPDR (E11.32X), moderate NPDR (E11.33X), severe NPDR (E11.34X) or PDR (E11.35X) in the past 5 years. General evaluation and management (E/M) codes (e.g., 992xx) were not used to define prior screening status, as they are utilized across non-ophthalmic medical specialties and do not reliably distinguish ophthalmic care. The code for type 2 diabetes without complications (E11.9) was not used as an indicator of prior screening because it is frequently used by non-ophthalmic providers and does not reliably indicate DR screening status. Those without these codes were assumed to have no prior known DR screening and were classified as the “no known history of DR screening” group. Figure 1 summarizes patient counts and cohort sizes at each stage of selection.

The flowchart illustrates cohort selection and stratification of patients with newly diagnosed proliferative diabetic retinopathy (PDR) requiring treatment. From the TriNetX Global Collaborative Network of 169,545,409 records, 10,141,607 patients aged 40 years or older with type 2 diabetes were identified. A total of 57,964 patients were identified with first-onset PDR requiring treatment. Patients were then stratified based on a known prior DR screening in the past 5 years, 32,961 (57%), and patients without a known prior screening, 25,003 (43%).

### 2.2. Covariates

Baseline demographics such as age at event, race, ethnicity and gender were recorded. Other diagnoses that have been previously associated with the risk of hospitalization were queried in both groups, including primary hypertension (I10), chronic ischemic heart disease (I25), heart failure (I50), diabetic circulatory complications (E11.5), diabetic nephropathy (E11.2), chronic kidney disease (N18), diabetic neuropathy (E11.4), peripheral neuropathy (G62.9), obesity (E66), hyperlipidemia (E78), tobacco use (Z72.0), nicotine dependence (F17), alcohol use disorder (F10), and mood disorders (F30–39) [8,17,18].

Next, propensity score matching (PSM) was performed on those with a known history of DR screening versus those with no known history of DR screening to control for age, gender, race, ethnicity, and the systemic comorbidities listed above. The PSM was performed using the TriNetX built-in analysis platform, which uses 1:1 matching by nearest neighbor greedy matching algorithm with a caliper of 0.25 standard deviations [22].

### 2.3. Outcome Measures

The primary outcomes, measured at 30, 60 and 90 days after the index event, included the rate of hospitalization as identified using visit information within TriNetX (CPT codes 1013659, 1013699, 1013729 or the SNOMED codes 394656005, 737481003, 86181006, 53923005), which has been demonstrated to be a sensitive and specific method of identifying hospitalization in prior TriNetX studies [8]. The follow-up intervals were defined to reflect real-world management of PDR requiring treatment as well as society guidelines. Patients receiving anti-VEGF therapy are typically followed at approximate 4-week intervals during the initial treatment phase. Society guidelines from the ADA suggest PDR be followed every 3 months, whereas the AAO recommends 2–4 months depending on the severity of disease, with shorter follow-ups in those with high-risk features requiring treatment. The rates of repeat ophthalmic examination were assessed using any repeat ICD-10 code for PDR (E11.352, E11.353, E11.354, E11.355, E11.359), which served as a proxy for an ophthalmic examination as well as repeat retinal imaging codes including OCT (CPT 92134), fundus photography (CPT 92250), and fluorescein angiography (CPT 92235, 92242).

## 3. Results

### 3.1. Baseline Characteristics

A total of 57,964 patients with newly diagnosed PDR without DME requiring treatment were identified. Of these, 32,961 (57%) patients had a known history of DR screening and 25,003 (43%) did not. Prior to propensity score matching (PSM), there were significant differences in the rate of all demographic characteristics and medical comorbidities (Table 1). The group with prior history of DR screening had higher rates of all comorbidities including primary hypertension (59.1% vs. 38.8%), hyperlipidemia (46.4% vs. 30.3%), chronic kidney disease (24.6% vs. 21.8%), obesity (21.4% vs. 13.2%), ischemic heart disease (17.7% vs. 15.0%), heart failure (15.3% vs. 11.5%), and mood disorders (13.6% vs. 7.7%). Similarly, this group had higher rates of diabetic complications such as diabetic nephropathy (23.7% vs. 18.6%), diabetic neuropathy (21.6% vs. 12.3%), and diabetic circulatory complications (9.1% vs. 6.1%). Notably, the group with known DR screening history also had higher mean HbA1c levels (8.38 ± 2.33 vs. 8.15 ± 2.25). After PSM methods were applied, a total of 19,316 patients were analyzed in each cohort (Table 1). Both groups were well-balanced across all comorbidities and diabetic complications with standardized differences <0.1, indicating successful matching [23,24].

### 3.2. Risk of Hospitalization

**Figure 2:**
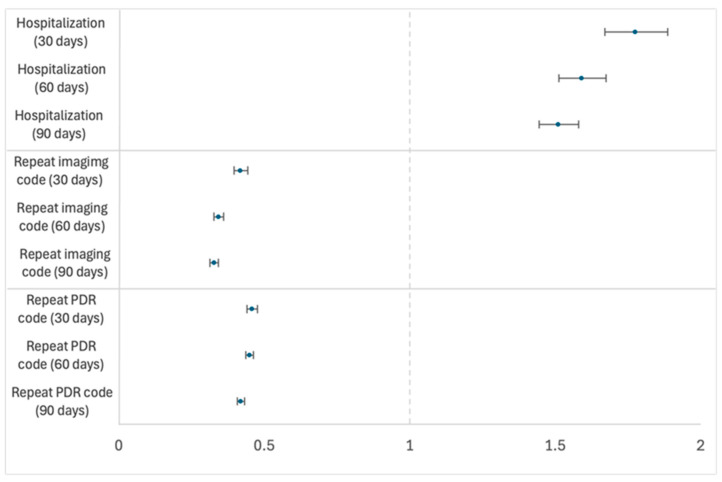
Forest plot illustrating risk ratios of hospitalization, repeat retinal imaging, and repeat PDR codes in PDR patients with and without known prior DR screening.

The cumulative rate of hospitalization was measured in each group 30, 60 and 90 days following the index event (Table 2). This rate was 13.3%, 17.5% and 20.3% in those without a known history of DR screening and 7.5%, 11.0% and 13.4% in those with a known history of DR screening at 30, 60 and 90 days, respectively. Patients without known DR screening history had significantly higher hospitalization rates with risk ratios (RRs) of 1.78 (95% CI 1.67–1.89), 1.59 (95% CI 1.51–1.67), and 1.51 (95% CI 1.44–1.58) at 30, 60, and 90 days respectively (all *p* < 0.0001; Figure 2).

The graph shows risk ratios (RRs) with 95% confidence intervals (CIs) for the risk of hospitalization, repeat retinal imaging, and repeat PDR codes in patients with newly diagnosed proliferative diabetic retinopathy (PDR) who had no known prior diabetic retinopathy (DR) screening compared to those with a known prior DR screening. The figure shows outcomes at 30, 60, and 90 days after PDR diagnosis and treatment initiation. An RR > 1 indicates an increased risk of hospitalization in patients without a known prior history of DR screening compared to those with a known prior DR screening. An RR < 1 demonstrates lower incidence of repeat retinal imaging or repeat PDR codes in patients without a prior DR screening compared to those with a known prior DR screening.

### 3.3. Rate of Repeat Visits

The cumulative rate of follow-up ophthalmic examination as estimated using ICD-10 PDR code was measured at 30, 60 and 90 days following the index event (Table 2). This rate was 16.2%, 25.3% and 26.8% in those without known history of DR screening and 35.3%, 56.2% and 62.5% in those with a known history of DR screening at 30, 60 and 90 days, respectively. The cumulative follow-up rate using this methodology was significantly lower in those without a known history of DR screening, with RRs of 0.458 (95% CI 0.440–0.476), 0.450 (95% CI 0.437–0.463), and 0.420 (95% CI 0.408–0.432) at 30, 60 and 90 days respectively (all *p* < 0.0001; Figure 2).

The cumulative rate of follow-up retinal examination as estimated using repeat retinal imaging CPT codes was measured 30, 60 and 90 days following the index event (Table 2). This rate was 8.5%, 12.4% and 14.2% in those without known history of DR screening and 20.3%, 36.3% and 43.2% in those with a known history of DR screening at 30, 60 and 90 days, respectively. The cumulative follow-up rate was significantly lower in those without a known history of DR screening, with RRs of 0.450 (95% CI 0.423–0.478), 0.394 (95% CI 0.377–0.411), and 0.381 (95% CI 0.366–0.396) at 30, 60, and 90 days, respectively (all *p* < 0.0001; Figure 2).

A secondary validation analysis was conducted using the unmatched cohort to ensure the findings were not artifacts of the PSM process. In this unadjusted analysis (Supplemental Table S2), patients without a known history of DR screening continued to show significantly higher rates of hospitalization at 30, 60, and 90 days after the index event, confirming that the increased hospitalization risk was not due to PSM methodology but represents a true association with screening history.

## 4. Discussion

In this large real-world study of patients with newly diagnosed PDR, patients without a known history of DR screening were up to 51–78% more likely to be hospitalized following diagnosis, with 20% of patients being hospitalized in the first 90 days. These patients were found to have a dramatically reduced rate of ophthalmic follow-up, with 54–62% fewer follow-up visits attended compared to those with a known history of DR screening.

These findings may be interrelated, as more frequent hospitalizations can lead to missed eye appointments, preventing patients from following up with ophthalmology and resulting in delays in retinal care. The major finding of the study is an increased rate of hospitalization in patients without a known history of DR screening, suggesting that lack of eye screening may be used as a proxy for broader gaps in healthcare engagement and disease control. While other forms of preventive care, such as primary care visits or routine metabolic monitoring, may also reflect healthcare engagement, we used prior DR screening as a diabetes-specific marker because it can be identified using established ophthalmic ICD-10 and CPT codes. However, this likely reflects only one aspect of overall healthcare utilization and may not fully capture a patient’s broader engagement with medical care. PDR often coexists with other end-organ complications of diabetes, with prior studies finding strong correlations with nephropathy, neuropathy, and cardiovascular disease as well as all-cause mortality [18]. In our combined PSM cohort, we found 44% of patients had concurrent hypertension, 20% had diabetic nephropathy and 15% had diabetic neuropathy [18]. Poor control of these conditions as well as underlying hyperglycemia increase the risk of hospitalization, with macrovascular complications, hyperglycemia, and infections being among the most common causes of hospitalization in diabetic patients [19].

Notably, prior to PSM, the cohort without a known history of DR screening had lower rates of nearly all documented comorbidities, which might initially suggest they were healthier. However, this is likely to reflect underdiagnosis rather than true absence of disease, as these patients had lower healthcare engagement overall. Despite having fewer documented comorbidities, our secondary validation analysis found that patients without known DR screening history had higher rates of hospitalization both before and after PSM, with the effect becoming even more pronounced after matching. This reinforces the concept that patients without prior screening may have more unrecognized or poorly controlled systemic conditions. In combination with other known risk factors, a lack of known DR screening history in a patient presenting with PDR could help ophthalmologists identify patients at higher risk of systemic complications. For such patients, expedited coordination with primary care providers and, when appropriate, urgent evaluation may help prevent decompensation and subsequent hospitalization. This is where ophthalmologists can play a crucial role in advancing public health, underscoring the importance of timely communication between medical specialists and primary care.

Another critical finding is the disparity in ophthalmic follow-up between both groups. Although PDR and retinal imaging codes are not a perfect metric for ophthalmic follow-up rates, they provide a method to estimate differences between groups. Prior studies have found that the most common presentation of type 2 DM patients with treatment-level PDR is vitreous hemorrhage, which is likely symptomatic, suggesting factors beyond awareness contributed to follow-up disparities [25,26]. Studies on the PDR population have found younger age and lower socioeconomic status are associated with higher rates of LTFU [8]. One study reported 25% LTFU over four years, with rates of LTFU as high as 30% in the African American population and 38% in the Hispanic population [8]. These findings were corroborated in a recent IRIS registry study which identified younger age and minority race/ethnicity as risk factors for LTFU [14]. Consistent with the literature, the cohort without a history of known DR in the current study was younger and had a higher proportion of minority patients prior to PSM; however, PSM allowed the study to account for these differences when assessing our primary outcomes.

Other logistical and financial barriers such as transportation challenges, inability to take time off work, and financial strain have been reported to contribute to LTFU but are not captured in billing databases such as TriNetX [10,11,12]. Additionally, unexpected hospitalizations may disrupt follow-up schedules and generate unforeseen expenses that further compound financial hardship. Prior reports have demonstrated how unexpected hospitalization can create a cascade of events that ultimately prevents patients from returning for ophthalmic care [22]. The findings of the current study suggest that lack of a known prior DR screening may serve as a proxy for identifying patients who are at greater risk of disengaging from follow-up, whether it be due to hospitalization or other barriers, even after a serious diagnosis.

The results of the current study carry implications for the management of PDR in the real-world. The advent of anti-VEGF therapy has provided an alternative to PRP, with Protocol S and CLARITY demonstrating excellent visual outcomes when intensive, regular treatment with proper compliance is maintained [6,27]. However, these regimens require frequent intravitreal injections and close monitoring, which may be unrealistic for patients at high-risk for LTFU who face real-world constraints. Even in the controlled environment of Protocol S, over 30% of participants were LTFU by year 5 [6]. Thus, when facing a patient with questionable reliability, clinicians may favor PRP to provide a more durable effect in case the patient does not return. This is supported by outcome data from patients diagnosed with PDR with LTFU. In an analysis of over 2600 patients that had interrupted care for more than 1 year, those initially treated with PRP fared better with fewer new complications and lower likelihood of severe vision loss compared to eyes managed with anti-VEGF alone [14]. It is worth noting that one study observed higher LTFU in patients receiving PRP as compared to injections [8]. One possibility for this observed difference may be selection bias, with clinicians opting for PRP in patients they anticipated would be less reliable. Alternatively, it may suggest that patients perceive PRP as a definitive treatment, thereby reducing their motivation to return to their clinic. Finally, it may also be that patients find PRP more unpleasant, which could discourage return visits. Regardless, the current study reinforces that patient reliability is a key factor to consider. In PDR patients where follow-up is less likely, timely well-performed PRP may be the safest strategy to prevent catastrophic vision loss.

A final important takeaway from the current study is that 43% of patients with first-time PDR diagnosis had no prior DR screening exam documented. This finding aligns with prior reports demonstrating persistent gaps in DR screening, and these issues are more common among patients with lower socioeconomic status or from minority populations. Reports studying these differences have identified limited healthcare access (particularly in rural areas), low health literacy, and breakdowns in care coordination as the major contributory issues [10,11,12]. They also report that younger patients and those from minority races/ethnicity are associated with lower diabetes screening rates [10,22]. Various interventions have sought to increase screening uptake by bringing eye exams directly to the patient’s point of care within primary or community settings; however, even with these programs, screening rates remain suboptimal at approximately 70% [15]. Importantly, these studies have found that the greatest barrier is not simply screening itself but ensuring effective linkage to follow-up eye care once retinopathy is detected [15]. The findings of the current study reinforce this concern and highlight how both access to screening and continued ophthalmic care are important gaps in the management of diabetic eye disease.

While the current study has several strengths including the use of a large, diverse, real-world dataset across multiple healthcare organizations that enhances the generalizability of the findings, it also has important limitations. Firstly, as with all billing databases, the electronic health record data is only as complete as what was recorded by the contributing health care organizations. It is possible that some patients categorized as no known history of DR screening did receive an eye exam at an eye institute that does not participate in the TriNetX network; however, this misclassification would tend to bias the results towards the null, so true differences could be even larger than observed. Additionally, the proxies to define prior DR screening were broadly set to maximize identification of prior eye care; however, some of these visits still may not have been captured. This could result in the misclassification of patients by classifying more patients as screened, biasing results toward the null. This can also affect the primary outcomes, as hospitalization and follow-up were only counted if the patient received care within the TriNetX network. The current study attempted to mitigate this by focusing on short follow-up intervals where cross-system migration is less likely; however, this remains a concern. Additionally, the reliance on ICD-10 and CPT codes as proxies for diagnoses and follow-up examinations may underestimate true follow-up rates if visits were not coded with PDR-specific diagnoses or if retinal examinations occurred without documentation of retinal imaging billing codes. To better capture follow-up care, the current study included multiple retinal imaging modalities; however, some follow-up visits may still not have been identified. Secondly, the study cannot capture granular details about the patients such as specific retinal findings or visual acuity, nor could it determine the specific reasons for hospitalization. The relationship between screening, follow-up and hospitalization is complex and likely influenced by many factors that are unmeasured within the billing database. Thirdly, this study did not directly measure visual outcomes or long-term vision loss, which are the most important endpoints for PDR; this was not performed in this study due to the high risk of attrition bias, as noted by the significantly lower rate of follow-up in the group without a known history of DR screening [14]. Finally, certain confounders including socioeconomic status, insurance coverage, and health literacy are not reliably captured in claims-based data. These factors may independently influence screening outcomes and hospitalization risk.

In conclusion, the current study demonstrates that diabetic patients that present with PDR without a known history of DR screening are at a higher risk of hospitalization and lower rate of follow-up with ophthalmic providers in the short-term period of 90 days. In patients diagnosed with PDR with a history of poor healthcare engagement, tailored strategies are needed to prevent vision loss as well as manage systemic disease.

## Figures and Tables

**Table 1:** Baseline characteristics of patients with newly diagnosed proliferative diabetic retinopathy (PDR) requiring treatment before and after propensity score matching (PSM).

	Before Matching				After Matching			
Characteristics	No Known Prior DR Screening (*N* = 25,003)	Known Prior DR Screening (*N* = 32,961)	*p*	Std. Diff.	No Known Prior DR Screening (*N* = 19,316)	Known Prior DR Screening (*N* = 19,316)	*p*	Std. Diff.
Age (yrs, mean ± SD)	59.5 ± 11.1	60.0 ± 10.9	<0.0001	0.0449	59.2 ± 11.1	59.1 ± 11.0	0.6085	0.0052
Sex								
Male	14,048 (56.19%)	17,673 (53.62%)	<0.0001	0.0516	10,683 (55.31%)	10,656 (55.17%)	0.7823	0.0028
Female	10,625 (42.50%)	15,177 (46.05%)	<0.0001	0.0715	8541 (44.22%)	8550 (44.26%)	0.9265	0.0009
Unknown	330 (1.32%)	111 (0.34%)	<0.0001	0.1086	92 (0.48%)	110 (0.57%)	0.2042	0.0129
Race								
White	8155 (32.62%)	15,832 (48.03%)	<0.0001	0.3182	7959 (41.20%)	8049 (41.67%)	0.3526	0.0095
Black or AA	3437 (13.75%)	7366 (22.35%)	<0.0001	0.2251	3421 (17.71%)	3460 (17.91%)	0.6040	0.0053
Asian	2713 (10.85%)	1313 (3.98%)	<0.0001	0.2643	1138 (5.89%)	1297 (6.72%)	0.0009	0.0339
Native Hawaiian or Other Pacific Islander	440 (1.76%)	405 (1.23%)	<0.0001	0.0438	340 (1.76%)	309 (1.60%)	0.2197	0.0125
American Indian or Alaskan Native	141 (0.56%)	260 (0.79%)	0.0012	0.0274	140 (0.73%)	135 (0.70%)	0.7622	0.0031
Unknown	8823 (35.29%)	5513 (16.73%)	<0.0001	0.4329	5075 (26.27%)	4800 (24.85%)	0.0013	0.0326
Other	1294 (5.18%)	2272 (6.89%)	<0.0001	0.0722	1243 (6.44%)	1266 (6.55%)	0.6349	0.0048
Ethnicity								
Not Hispanic or Latino	10,424 (41.69%)	16,203 (49.16%)	<0.0001	0.1504	8809 (45.61%)	8987 (46.53%)	0.0692	0.0185
Hispanic or Latino	3868 (15.47%)	9326 (28.29%)	<0.0001	0.3140	3862 (19.99%)	3973 (20.57%)	0.1602	0.0143
Unknown	10,711 (42.84%)	7432 (22.55%)	<0.0001	0.4430	6645 (34.40%)	6356 (32.91%)	0.0019	0.0317
Comorbidities (ICD-10)								
HTN (I10)	9698 (38.79%)	19,488 (59.12%)	<0.0001	0.4155	8800 (45.56%)	8514 (44.08%)	0.0034	0.0298
HLD (E78)	7582 (30.32%)	15,294 (46.40%)	<0.0001	0.3352	6848 (35.45%)	6663 (34.50%)	0.0484	0.0201
CKD (N18)	5455 (21.82%)	8116 (24.62%)	<0.0001	0.0665	4435 (22.96%)	4259 (22.05%)	0.0320	0.0218
Diabetic Nephropathy (E11.2)	4638 (18.55%)	7816 (23.71%)	<0.0001	0.1267	3966 (20.53%)	3778 (19.56%)	0.0169	0.0243
Overweight/Obese (E66)	3298 (13.19%)	7064 (21.43%)	<0.0001	0.2191	3123 (16.17%)	3102 (16.06%)	0.7714	0.0030
T2DM w/neurological complications (E11.4)	3068 (12.27%)	7108 (21.57%)	<0.0001	0.2498	2867 (14.84%)	2813 (14.56%)	0.4379	0.0079
Chronic Ischemic Heart Disease (I25)	3758 (15.03%)	5830 (17.69%)	<0.0001	0.0719	3169 (16.41%)	2958 (15.31%)	0.0033	0.0299
Heart Failure (I50)	2875 (11.50%)	5043 (15.30%)	<0.0001	0.1118	2535 (13.12%)	2433 (12.60%)	0.1211	0.0158
Mood/Affective Disorders (F30-F39)	1935 (7.74%)	4475 (13.58%)	<0.0001	0.1900	1822 (9.43%)	1737 (8.99%)	0.1348	0.0152
T2DM w/circulatory complications (E11.5)	1532 (6.13%)	3010 (9.13%)	<0.0001	0.1134	1401 (7.25%)	1326 (6.87%)	0.1363	0.0152
Nicotine dependence (F17)	1189 (4.76%)	2643 (8.02%)	<0.0001	0.1337	1123 (5.81%)	1037 (5.37%)	0.0569	0.0194
Polyneuropathy (G62.9)	829 (3.32%)	2133 (6.47%)	<0.0001	0.1467	795 (4.12%)	758 (3.92%)	0.3379	0.0098
Alcohol Dependence or Abuse (F10)	384 (1.54%)	840 (2.55%)	<0.0001	0.0716	358 (1.85%)	338 (1.75%)	0.4443	0.0078
Current Tobacco Use (Z72.0)	380 (1.52%)	823 (2.50%)	<0.0001	0.0697	362 (1.87%)	318 (1.65%)	0.0887	0.0173
Lab Values								
Hgb A1C (mean ± SD)	8.15 ± 2.25 *N* = 8257	8.38 ± 2.33 *N* = 13,694	<0.0001	0.1031	8.19 ± 2.28 *N* = 6840	8.30 ± 2.24 *N* = 6738	0.6733	0.0104

Patient demographics, race/ethnicity, and comorbidities are presented for both groups stratified by known history of diabetic retinopathy (DR). Standardized differences are shown for balance before and after PSM. AA = African American; HTN = Hypertension; HLD = Hyperlipidemia; CKD = Chronic Kidney Disease; T2DM = Type 2 Diabetes Mellitus; Hgb A1C = Hemoglobin A1C; SD = Standard Deviation; Std. Diff. = Standard Difference.

**Table 2:** Short-term hospitalization and ophthalmology follow-up outcomes with PSM.

Outcome	Time Period	No Known Prior DR Screening	Known Prior DR Screening	Risk Ratio	95% CI	*p*
Hospitalization	30 days	2574 (13.322%)	1450 (7.505%)	1.775	1.670–1.887	<0.0001
	60 days	3378 (17.484%)	2123 (10.988%)	1.591	1.513–1.674	<0.0001
	90 days	3913 (20.253%)	2590 (13.405%)	1.511	1.444–1.581	<0.0001
Repeat imaging code	30 days	1645 (8.515%)	3923 (20.305%)	0.419	0.396–0.444	<0.0001
	60 days	2404 (12.443%)	7004 (36.253%)	0.343	0.327–0.360	<0.0001
	90 days	2740 (14.182%)	8348 (43.209%)	0.329	0.313–0.342	<0.0001
Repeat PDR code	30 days	3121 (16.158%)	6820 (35.309%)	0.458	0.440–0.476	<0.0001
	60 days	4887 (25.302%)	10,859 (56.221%)	0.450	0.437–0.463	<0.0001
	90 days	5072 (26.777%)	12,077 (62.527%)	0.420	0.408–0.432	<0.0001

Outcomes are reported at 30, 60, and 90 days following proliferative diabetic retinopathy (PDR) diagnosis and treatment. Risk ratios (RRs) with 95% confidence intervals (CIs) compare patients with no known DR screening in the prior 5 years to those with a known prior DR screening. PSM = Propensity Score Matching; DR = Diabetic Retinopathy; PDR = Proliferative Diabetic Retinopathy.

## Data Availability

The data presented in this study are available on request from TriNetX (https://trinetx.com/). Restrictions apply to the availability of these data, which were accessed under license for this study. Data access requests can be directed to the corresponding author.

## Supplementary Materials

The following supporting information can be downloaded at: https://www.mdpi.com/article/10.3390/diagnostics16101562/s1, Table S1: Codebook of ICD-10, CPT, and SNOMED codes. Used for Cohort Identification and Outcomes. Table S2: Short-term hospitalization outcomes without propensity score matching (PSM).
