# Supplementary material for: Impact of Prior Diabetic Retinal Screening on Hospitalization and Ophthalmic Follow-Up in Diabetic Patients with Newly Diagnosed Proliferative Diabetic Retinopathy
Source: Diagnostics (Basel). 2026 May 21;16(10):1562. doi: 10.3390/diagnostics16101562 (PMC13205404; doi:10.3390/diagnostics16101562)
Supplement: Supplementary file 1 [file diagnostics-16-01562-s001.zip › Supplemental Table S1.pdf]

Supplemental Table S1. Codebook of ICD-10, CPT, and SNOMED Codes Used for Cohort Identification and Outcomes.

Category	Codes Used	Description
**Diabetes Identification**	E11.X (exclude E10.X, E13.X)	Type 2 Diabetes Mellitus
**Hemoglobin A1c Inclusion**	A1c ≥ 6.5%	Alternate diabetes confirmation
**PDR Identification**	E11.352, E11.353, E11.354, E11.355, E11.359	Proliferative Diabetic Retinopathy
**Treatment Procedures**	67228	Pan-retinal Photocoagulation
	67028	Intravitreal Injection
**Imaging**	92134	Optical Coherence Tomography
**Vitreous Hemorrhage**	H43.1	Vitreous Hemorrhage
**Exclusion for Vitreous Hemorrhage Algorithm**	H43.81, S05.X, H34.81, H34.82	Posterior Vitreous Detachment, Eye Injury, Retinal Vein Occlusion
**Prior DR Screening (DM Screen Codes)**	E11.32, E11.33, E11.34, E11.35	Non-Proliferative and Proliferative DR Screening
**Comorbidities - Hypertension**	I10	Primary Hypertension
**Comorbidities - CAD**	I25	Chronic Ischemic Heart Disease
**Comorbidities - Heart Failure**	I50	Heart Failure
**Comorbidities - Circulatory Complications**	E11.5	Diabetic Circulatory Complications
**Comorbidities - Nephropathy**	E11.2	Diabetic Nephropathy
**Comorbidities - Chronic Kidney Disease**	N18	Chronic Kidney Disease

Category	Codes Used	Description
**Comorbidities - Neuropathy**	E11.4, G62.9	Diabetic Neuropathy; Peripheral Neuropathy
**Comorbidities - Obesity**	E66	Obesity
**Comorbidities - Hyperlipidemia**	E78	Hyperlipidemia
**Comorbidities - Tobacco Use**	Z72.0, F17	Tobacco Use; Nicotine Dependence
**Comorbidities - Alcohol Use**	F10	Alcohol Use Disorder
**Comorbidities - Mood Disorders**	F30-F39	Mood Disorders
**Hospitalization Identification**	CPT: 1013659, 1013699, 1013729; SNOMED: 394656005, 737481003, 86181006, 53923005	Inpatient Hospitalization Codes
**Complications - TRD**	E11.352, E11.353, E11.354, H33.4x	Tractional Retinal Detachment
**Complications - Vitrectomy**	67036, 67040, 67041, 67042, 67113	Vitrectomy Procedures
**Complications - Neovascular Glaucoma**	H40.5, H21.1	Neovascular Glaucoma
**Complications - Low Vision / Blindness**	H54.0, H54.1, H54.2, H54.4, H54.5, H54.8	Blindness and Low Vision

ICD-10 = International Classification of Diseases, 10th Revision; CPT = Current Procedural Terminology; SNOMED = Systematized Nomenclature of Medicine Clinical Terms.
