# Supplementary material for: Impact of Prior Diabetic Retinal Screening on Hospitalization and Ophthalmic Follow-Up in Diabetic Patients with Newly Diagnosed Proliferative Diabetic Retinopathy
Source: Diagnostics (Basel). 2026 May 21;16(10):1562. doi: 10.3390/diagnostics16101562 (PMC13205404; doi:10.3390/diagnostics16101562)
Supplement: Supplementary file 1 [file diagnostics-16-01562-s001.zip › Supplemental Table S2.pdf]

Supplemental Table S2. Short-term hospitalization outcomes in patients with and without known history of prior DR screening *without* PSM.

Outcome	Time Period	No Known Prior DR Screening (N = 25,003)	Known Prior DR Screening (N = 32,961)	Risk Ratio	95% CI	P
Hospitalization	30 days	2847 (11.389%)	2649 (8.037%)	1.417	(1.34-1.498)	<0.0001
	60 days	3853 (15.410%)	4026 (12.214%)	1.262	(1.207-1.319)	<0.0001
	90 days	4531 (18.122%)	5002 (15.176%)	1.194	(1.147-1.242)	<0.0001

Outcomes are reported at 30, 60, and 90 days following proliferative diabetic retinopathy (PDR) diagnosis and treatment. Risk ratios (RR) with 95% confidence intervals (CIs) compare patients with no recent DR screening to those with recent screening.

DR = Diabetic Retinopathy; PSM = Propensity Score Matching.
